# Molecular Characterization of Gastric Carcinoma: Therapeutic Implications for Biomarkers and Targets

**DOI:** 10.3390/biomedicines6010032

**Published:** 2018-03-09

**Authors:** Lionel Kankeu Fonkoua, Nelson S. Yee

**Affiliations:** 1 Department of Medicine, Penn State Health Milton S. Hershey Medical Center, Hershey, PA 17033, USA; lkankeufonkoua@pennstatehealth.psu.edu; 2 Division of Hematology-Oncology, Department of Medicine, Penn State Health Milton S. Hershey Medical Center, Experimental Therapeutics Program, Penn State Cancer Institute, Pennsylvania State University College of Medicine, Hershey, PA 17033, USA

**Keywords:** Asian Cancer Research Group (ACRG), gastric carcinoma, molecular profiling, precision therapy, pembrolizumab, predictive biomarkers, ramucirumab, The Cancer Genome Atlas (TCGA), therapeutic targets, trastuzumab

## Abstract

Palliative chemotherapy is the mainstay of treatment of advanced gastric carcinoma (GC). Monoclonal antibodies including trastuzumab, ramucirumab, and pembrolizumab have been shown to provide additional benefits. However, the clinical outcomes are often unpredictable and they can vary widely among patients. Currently, no biomarker is available for predicting treatment response in the individual patient except human epidermal growth factor receptor 2 (HER2) amplification and programmed death-ligand 1 (PD-L1) expression for effectiveness of trastuzumab and pembrolizumab, respectively. Multi-platform molecular analysis of cancer, including GC, may help identify predictive biomarkers to guide selection of therapeutic agents. Molecular classification of GC by The Cancer Genome Atlas Research Network and the Asian Cancer Research Group is expected to identify therapeutic targets and predictive biomarkers. Complementary to molecular characterization of GC is molecular profiling by expression analysis and genomic sequencing of tumor DNA. Initial analysis of patients with gastroesophageal carcinoma demonstrates that the ratio of progression-free survival (PFS) on molecular profile (MP)-based treatment to PFS on treatment prior to molecular profiling exceeds 1.3, suggesting the potential value of MP in guiding selection of individualized therapy. Future strategies aiming to integrate molecular classification and profiling of tumors with therapeutic agents for achieving the goal of personalized treatment of GC are indicated.

## 1. Introduction

With about a million diagnosed cases and over 700,000 deaths recorded annually, gastric carcinoma (GC) is the third most common cause of cancer deaths worldwide [1]. While 80 to 90% of tumors develop sporadically, hereditary factors also contribute to gastric carcinogenesis [2]. The incidence is strongly influenced by ethnicity, diet, and infectious agents [3,4,5,6]. In particular, *Helicobacter pylori* (*H. pylori*) and human papilloma virus (HPV) are involved in multi-step processes causing chronic gastritis, intestinal metaplasia, and invasive carcinoma [7,8,9]. Population-based screening and treatment of *H. pylori* and HPV would be a logical strategy for prevention of some types of GC, but no randomized trial to date has shown a clear benefit of this approach [10]. Until a preventive intervention is implemented, it is imperative that effective and tolerable therapies are developed in attempt to attenuate the global burden of GC.

Systemic chemotherapy and targeted therapy play important roles in the multi-disciplinary management of GC. With the exception of GC diagnosed at T1 stage, chemotherapy is employed in the neoadjuvant and adjuvant settings, or concurrent with radiation therapy. Palliative combination chemotherapy and targeted therapy are the only treatment options for patients with advanced or metastatic GC. Selection of chemotherapeutic drugs is typically based on performance status, medical comorbidities, and medical oncologist’s experience or preference. There are no valid biomarkers predictive of treatment response of GC to therapeutic agents. Exceptions are, amplification of human epidermal growth factor receptor 2 (HER2) and expression of programmed death-ligand 1 (PD-L1), for which trastuzumab and pembrolizumab, respectively, have been demonstrated to produce clinical benefit [11,12]. Preliminary evidence has indicated that variable responses to treatment can be attributed to tumor heterogeneity with regard to molecular alterations [13]. Recently, two classification systems of GC using multi-platforms of molecular analyses have been developed, and they provide new insights into tumor heterogeneity of GC.

The genomic characterization of GC has led to the development of two new classifications of GC by The Cancer Genome Atlas (TCGA) Research Network [14] and the Asian Cancer Research Group (ACRG) [15]. These may serve as a valuable diagnostic companion to the conventionally used classification systems of GC based on histopathology by World Health Organization [16] and Lauren [17]. Importantly, TCGA and ACRG are expected to facilitate the development of personalized prognostication and treatment, as well as improved patient stratification for clinical trial design. Moreover, molecular profiling of GC has been accomplished through immunohistochemistry (IHC), in situ hybridization (ISH), genomic DNA sequencing, proteomics, and microRNA expression. The tumor molecular profiles can potentially be developed into predictive biomarkers of treatment that could help guide selection of cytotoxic drugs and targeted therapeutics.

The goal of this article is to provide a critical review of the molecular characterization of GC, and elaborate on the molecular features that can be translated into therapeutic biomarkers and targets for clinical use. First, we provide an overview of the conventionally used systemic chemotherapy and targeted therapeutics of GC. The data on molecular classification of GC by TCGA and ACRG as well as molecular profiling of GC are examined. The potential of translating the molecular classification and profiling of GC into therapeutic targets and predictive biomarkers are discussed. We hope that this article will help identify the opportunity and challenge of developing strategies towards the goal of precision medicine in GC by improving therapeutic efficacy and minimizing treatment-related toxicity.

## 2. Systemic Treatment of Gastric Carcinoma

Systemic chemotherapy is employed for treatment of patients with localized GC as well as for those with advanced GC. Surgical resection with pre- and post-operative chemotherapy and/or radiation therapy represents the primary curative treatment of early-stage GC with 5-year survival rate of less than 30% [18,19,20]. For patients with advanced unresectable or metastatic disease, palliative systemic therapy and chemoradiation therapy are the standard treatment options. The chemotherapeutic regimens used for patients with advanced or metastatic GC are essentially the same as those for peri-operative treatment of patients with localized GC. In addition, for advanced or metastatic GC, trastuzumab is indicated to use in combination with HER2 amplified GC as first-line treatment; ramucirumab either as monotherapy or in combination with paclitaxel is indicated as second-line treatment; pembrolizumab has recently been approved as 3rd-line treatment for GC expressing PD-L1. A number of targeted therapeutics is being investigated in clinical studies.

### 2.1. Chemotherapy

Chemotherapeutic regimens currently being used for GC consist of anthracycline, fluoropyrimidine, taxane, and platinum-based agents. For advanced or metastatic GC, first-line combinations such as EOX (epirubicin, oxaliplatin, capecitabine) and DCF (docetaxel, cisplatin, 5-fluorouracil (5-FU) have produced limited survival benefits, with median survival not exceeding one year (Table 1). Second-line agents such as docetaxel or irinotecan can lead to slight improvement of survival. For most patients, GC may initially respond to chemotherapy. However, the tumors will typically become resistant, such that the prognosis of patients with advanced disease remains poor. Currently, there is no clinically available predictor of tumor response to the empiric use of these drug combinations [21].

### 2.2. Targeted Therapy

Despite the clinical heterogeneity and molecular complexity of GC, targeted therapeutics directed against the genetic mutations and signaling pathways that drive tumor growth and invasion have been developed and clinically investigated. Targeted therapies currently in clinical use include trastuzumab, ramucirumab, and pembrolizumab. Other targeted therapeutics directed against the signaling pathways of mitogenesis, angiogenesis, and immune checkpoints are under clinical investigation for treatment of GC.

#### 2.2.1. Mitogenic Signaling Pathways as Therapeutic Targets

Human epidermal growth factor receptor (HER2, also known as ErbB2) is a transmembrane tyrosine kinase receptor of the ErbB family. The ErbB members play important roles in regulation of cellular functions including proliferation, growth, survival, adhesion, migration, and differentiation. HER2 acts by heterodimerization with other ErbB family receptors leading to activation of the RAS-MAPK (mitogen-activated protein kinase) and PI3K-AKT (phosphatidylinositol 3-kinase—AKT) pathways. HER2 has been found to be over-expressed in 20% to 30% of GC depending on the tumor subtype and location.

Several HER2-targeting agents have been developed and evaluated in phase III trials for patients with advanced HER2-positive gastroesophageal junction carcinoma (GEJC)/GC. Trastuzumab is a humanized IgG1 monoclonal antibody (mAb) directed against the extracellular domain of HER2, and it prevents dimerization of the HER2 receptors. This triggers receptor internalization and mediates antibody-dependent cell-mediated cytotoxicity (ADCC), resulting in inhibition of tumor growth [26]. In the phase III ToGA trial, the combination of trastuzumab and cisplatin/fluoropyrimidine-based chemotherapy was compared to chemotherapy alone as first-line therapy for advanced HER2-positive GEJC/GC. Results of this study indicated a significant improvement in the overall response rate (ORR; 47% vs. 35%; *p* < 0.01) as well as prolonged progression-free survival (PFS; 6.7 months vs. 5.5 months, *p* < 0.01) and overall survival (OS; 13.8 months vs. 11.1 months; *p* < 0.01) [11]. Based on the results of this study, the combination of trastuzumab with platinum/fluoropyrimidine-based chemotherapy has become the standard of care for advanced HER2-positive GEJC/GC. However, the treatment response was not durable, as the benefit from trastuzumab was noted to have diminished considerably in an updated survival analysis, with an increased hazard ratio (HR) from 0.73 to 0.80, and narrowed OS difference to 1.4 months. These data suggest considerable heterogeneity among patients with HER2-positive GEJC/GC, possible treatment resistance, and need to refine and optimize biomarker selection criteria for future clinical trials. A recent report shows that trastuzumab conjugated with nanoparticle albumin-bound paclitaxel produced enhanced anti-tumor effect in a mouse xenograft of HER2-positive gastric cancer cells [27]. Further investigation of this antibody-nanoparticle conjugate in patients may raise hope for a novel form of targeted therapy in HER2-positive GC.

#### 2.2.2. Signaling Pathways in Angiogenesis as Therapeutic Targets

Several signaling pathways are involved in tumor-associated angiogenesis, such as those activated by vascular endothelial growth factor (VEGF) [28], angiopoietins and angiopoietin-like proteins [29,30], platelet-derived growth factor (PDGF) [31], basic fibroblast growth factor (FGF) [32], fibroblast activation protein and hepatocyte growth factor [33], and Wingless-related integration site (WNT) [34]. These growth factors and their receptors have been investigated for therapeutic targeting in various types of malignant tumors. Antibodies directed against VEGF and VEGF receptor (VEGFR) have been shown to produce anti-tumor efficacy and they are used in combination with cytotoxic chemotherapy as standard first- or second-line treatment of certain solid tumors. 

VEGF is a growth factor secreted by the tumor to stimulate formation of new blood vessels in response to hypoxia and nutrient depletion. When it binds to VEGFR, a complex cascade of downstream signaling pathways is activated, resulting in neovascularization, vasodilation, and increased vascular permeability [28]. Blockade of VEGF and/or VEGFR impedes these pathways and thereby inhibits tumor survival, migration, and invasion. VEGF and its receptors are over-expressed in approximately 30% to 40% of all GEJC/GC [35,36], and anti-angiogenic agents targeting VEGF and VEGFR have shown therapeutic efficacy in GEJC/GC.

Ramucirumab is a recombinant humanized monoclonal antibody (mAb) that binds to VEGF-R2 and prevents its activation by VEGF. In contrast to bevacizumab (anti-VEGFA mAb), it has shown clinical efficacy as a single agent (REGARD trial) and in combination with paclitaxel (RAINBOW trial). Based on the results of these studies, ramucirumab either alone or in combination with paclitaxel has become standard second-line treatments for advanced GEJC/GC. In the REGARD trial, ramucirumab was associated with a significant improvement in OS (5.2 months vs. 3.8 months, *p* = 0.0473) and PFS (2.1 months vs. 1.3 months, *p* < 0.0001) in patients previously treated with first-line platinum- or fluoro-pyrimidine-based therapy [37]. In the RAINBOW study, the combination of ramucirumab and paclitaxel produced significant improvement in OS (9.6 months vs. 7.4 months, *p* = 0.0169), PFS (4.4 months vs. 2.9 months, *p* < 0.0001), and ORR (28% vs. 16%, *p* = 0.0001) compared with those treated with paclitaxel alone [38]. However, the clinical benefit of ramucirumab with or without paclitaxel is limited, and there is no biomarker available to predict tumor response to these treatments.

#### 2.2.3. Immune Checkpoint Molecules as Targets for Therapy

The programmed death-ligand 1 (PD-L1) and 2 (PD-L2) are normally expressed on antigen-presenting cells (APC) and also on tumor cells. Binding of PD-L1 and PD-L2 to their receptors (PD-1) on activated T cells leads to downregulation of cytotoxic T-cell activity and causing immunosuppression. PD-L1 is expressed in 15% to 70% of GCs, and they are associated with poor prognosis [39]. Pembrolizumab and nivolumab are humanized mAbs directed against PD-1, and they enhance the ability of the immune system to detect and destroy cancer cells. By blocking the interaction between PD-1 and PD-L1/L2, pembrolizumab or nivolumab counters the tumor’s immune-escaping tactic.

In the phase Ib KEYNOTE-012 trial, the activity and safety of pembrolizumab were evaluated in a cohort of 39 patients with advanced GEJC/GC. Pembrolizumab produced an ORR of 22.2%, 6-month PFS rate 24%, and OS rate 69% [40,41]. An association between higher levels of PD-L1 expression and ORR (*p* = 0.102), PFS (*p* = 0.162), and OS (*p* = 0.124) was observed. In a phase II study (KEYNOTE-059) of 259 patients, pembrolizumab monotherapy showed clinical efficacy in previously treated advanced GEJC/GC [12]. For patients with PD-L1 positive tumors, pembrolizumab produced an ORR of 15.5%, whereas in patients with PD-L1 negative tumors, 6.4%. In patients with microsatellite-high (MSI-H), ORR was 57.1%; in those with non-MSI-H tumors, ORR 9.0%. These data demonstrate the value of PD-L1 and MSI-H as predictive biomarkers for efficacy of pembrolizumab. In the cohort 2 of this study, the efficacy and safety of pembrolizumab in combination with cisplatin and 5-fluorouracil (5-FU) will be assessed.

## 3. Molecular Classification and Profiling of Gastric Carcinoma

Advances in next-generation sequencing (NGS) technologies and improved understanding of cancer biology have unlocked opportunities to characterize the genomic landscape of cancer including GC. Using multi-platform analyses, molecular profiling of GC has enabled The Cancer Genome Atlas (TCGA) Research Network and the Asian Cancer Research Group (ACRG) to classify GC into subtypes. The new molecular classification of GC is complementary to the conventionally used system of subtyping GC based on histopathology. Importantly, molecular classification of GC helps identify molecular alterations that may be targeted for therapy. Furthermore, molecular profiling of GC collected from individual patients using a multi-platform approach has offered new opportunity to identify biomarkers that may be predictive of tumor response to treatment [42,43,44].

### 3.1. TCGA Sub-Typing of Gastric Carcinoma: Potential Therapeutic Targets

**Figure 1:**
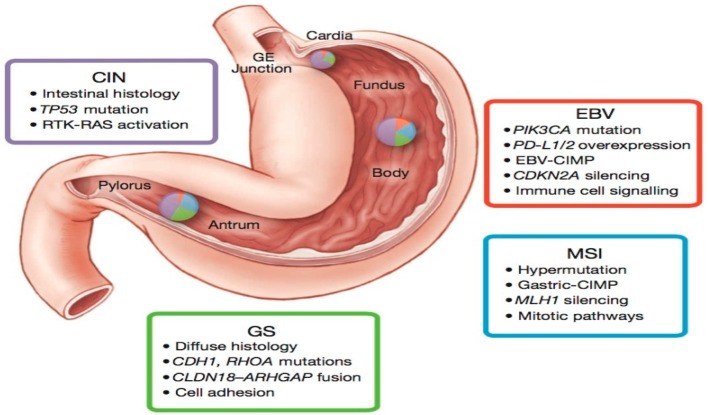
The four molecular subtypes described in the TCGA study, their mutational patterns, and location. CIN, chromosomal instability; EBV, Epstein–Barr virus; GE, gastroesophageal junction; GS, genomically stable; MSI, microsatellite instability. This figure is reproduced from reference [14] with permission from the Nature Publishing Group.

Molecular classification of GC by the TCGA Research Network utilized six distinct platforms, including exome sequencing, copy number analysis, methylation, expression of miRNA and mRNA. Based on TCGA molecular data, GC were divided into four groups: Epstein–Barr virus-positive (EBV; 9%), microsatellite instability (MSI; 22%), chromosomal instability (CIN; 50%), genomically stable (GS; 20%) (Figure 1). Each of these GC subtypes is characterized by distinct features that provide prognostic information and suggest potential benefit of targeted therapy.

The EBV-positive tumors were found to be mainly located in the gastric fundus or body. They exhibited higher prevalence of DNA promoter hypermethylation, A to C transversions, *PIK3CA* mutation, recurrent JAK2 and ERBB2 amplifications, interleukin-12 (IL-12) mediated signaling, and PD-L1/2 overexpression. The presence of viral antigens such as EBV (a hallmark of 9% of GCs) has been shown to result in increased neo-epitope presentation [14], which might contribute to an anti-tumor immune response. Moreover, the strength of IL-12 mediated signaling signature suggests a robust immune cell presence, which when coupled with evidence of PD-L1/2 overexpression, provides support for targeted immunotherapy. PD-L1/2 may therefore represent promising targets in these tumors and initial promising results have been reported with pembrolizumab [40,41]. In addition, the strong predilection for mutation in *PIK3CA* (80%) suggests that inhibition of PI3K warrants further evaluation in EBV-positive GC.

The MSI tumors, characterized by genomic instability due to a deficient DNA mismatch repair system, lacked targetable amplifications. This subtype of tumors was noted to have hypermethylation in the *MLH1* promoter region (leading to MLH1 silencing), and targetable hotspot mutations in *PIK3CA*, *ERBB3*, *ERBB2*, and *EGFR*. Of note, the *BRAF^V600E^* mutation commonly seen in MSI colorectal cancer was absent. However, gastric MSI tumors had a high rate of PD-L1 expression. In particular, recent evidence shows that enhanced anti-PD-1 responsiveness of mismatch repair-deficient tumors is related to the high number of mutation-associated neoantigens [45].

The CIN tumors were more frequent in the gastro-esophageal junction/cardia. They were noted to have the highest frequency of *TP53* mutations (71%), as well as genomic amplifications of RTKs and cell cycle mediators. Phosphorylation of EGFR was significantly elevated. Recurrent amplification of the gene encoding ligand VEGFA was also notable. Additionally, frequent amplifications of cell cycle mediators (*CCNE1*, *CCND1* and *CDK6*) were present. Alterations of these genes have been confirmed in a cohort of 116 advanced/metastatic GC cases [44].

The GS tumors, which lack either chromosomal alteration or microsatellite instability, exhibited elevated expression of molecules in the cell adhesion and angiogenesis-related pathways. Previous studies had demonstrated loss of the tumor-suppressor gene *CDH1* encoding the cell adhesion molecule E-cadherin in hereditary diffuse gastric cancer [6]. The TCGA data also revealed recurrent mutations in *RHOA* (Ras homolog gene family, member A) and fusion of *CLDN18-ARHGAP6* or 26 (30% of cases). RHOA modulates programmed cell death and actomyosin-dependent cell contractility and motility [46,47,48], while *CLDN18* and *ARHGAP6* are involved in intercellular tight junction structure and Rho signaling activation, respectively. Thus, alterations in either *RHOA* or *CLDN18-ARHGAP6* might contribute to lack of cellular cohesion, dispersed growth, and resistance to programmed cell death.

The TCGA data indicate that each of the four defined molecular subtypes displays distinct but overlapping candidate therapeutic targets. These suggest the potential of targeted therapeutics in each subtype of GC (Table 2). The discovery of mutations in the *RHOA* and *CLDN18* gene products could be exploited to develop new therapeutic strategies in the genomically stable subtype.

### 3.2. ACRG Sub-Typing of Gastric Carcinoma: Potential Prognostic Biomarkers

**Figure 2:**
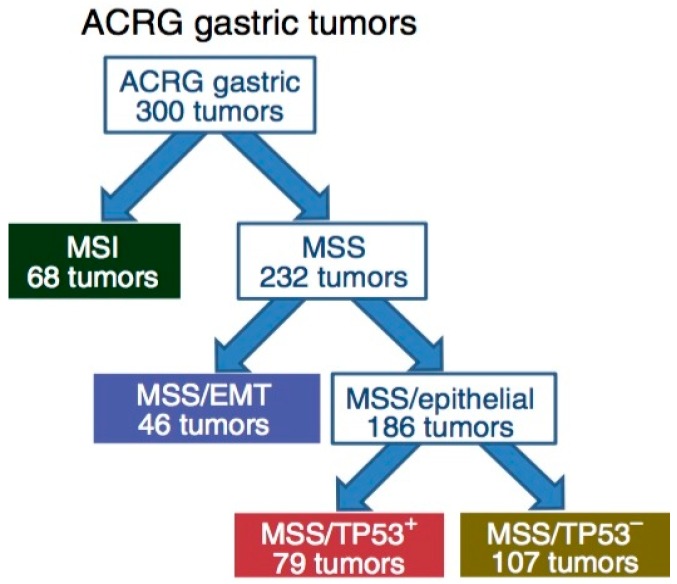
Illustration of the Asian Cancer Research Group (ACRG) classification tree. EMT, epithelia-to-mesenchymal transition; MSI, microsatellite instability; MSS, microsatellite stability. This figure is reproduced from reference [15] with permission from the Nature Publishing Group.

Complementary to the TCGA data, the ACRG proposed a classification of GC that correlates four molecular subtypes with distinct patterns of molecular alterations, disease progression and prognosis. The molecular analyses include principal component analysis (PCA) [49] of expression data and compared the association of the first three principal components with a small pre-defined set of gene expression signatures relevant to biology of GC. These include epithelial-to-mesenchymal transition (EMT) [50], microsatellite instability (MSI) [51], cytokine signaling [52], cell proliferation [53], DNA methylation [54], p53 activity [55], and gastric tissue [56]. Of the 300 specimens of GC being analyzed, the MSI subtype accounts for 23%, MSS/EMT 20%, MSS/TP53^+^ (mutated) 26%, and MSS/TP53^−^ (wild-type) 36% (Figure 2). *TP53* is the most frequently mutated gene in GC, and the status of p53 activation is based on a two-gene (CDKN1A and MDM2) p53-activity signature. 

The MSI tumors, as in the TCGA cohort, were found to be hypermutated [57,58] intestinal-subtype tumors occurring in the antrum. It is associated with the best overall prognosis and the lowest frequency of recurrence (22%) of the four subtypes. They exhibited mutations in genes such as *KRAS* (23.3%), the PI3K-PTEN-mTOR pathway (42%), *ALK* (16.3%) and *ARID1A* (44.2%) [46].

The MSS/TP53^+^ phenotype is associated with a better prognosis, and a higher prevalence of mutations in *APC*, *ARID1A*, *KRAS*, *PIK3CA* and *SMAD4*, compared to the MSS/TP53^−^ phenotype. Consistent with these observations, mutations in *TP53* (54%), *APC* (10%), *SMAD4* (5.9%), *KRAS* (5.9%), and *PIK3CA* (5.1%) were present at a high rate in a large cohort of 666 specimens of GC [42]. The MSS/TP53^−^ phenotype exhibited the highest prevalence of *TP53* and *RHOA* mutations, as well as recurrent focal amplifications in *ERBB2*, *CCNE1* and *CCND1*.

The MSS/EMT phenotype includes tumors of the diffuse-subtype. It is associated with the worst prognosis, tendency to occur at an earlier age, and the highest recurrence frequency (63%) of the four subtypes. The MSS/EMT subtype also includes a large set of signet ring cell carcinomas and showed loss of *CDH1* expression.

The ACRG subtyping of GC could be complementary to the TCGA system for molecular classification of GC. The ACRG data are potentially important for generating prognostic biomarkers in GC. The validity of these biomarkers for prognosis of patients with GC will need to be investigated in prospective clinical studies.

### 3.3. Comparison of TCGA and ACRG Data

TCGA and ACRG integrated the results of a wide scale molecular analysis into two different and partially overlapping models encompassing four molecular subtypes with distinct salient genomic features (Table 3). They both identified a MSI subtype characterized by high mutation frequency and best prognosis. While CIN and GS TCGA subtypes tumors were present across all ACRG subtypes, TCGA GS, EBV^+^, and CIN subtypes were enriched in ACRG MSS/EMT, MSS/TP53^+^, and TP53^−^ subtypes, respectively. However, CDH1 and RHOA mutations were highly prevalent in the TCGA GS subtype but infrequent in the ACRG MSS/EMT subtype, these two subtypes were deemed not equivalent. Similarly, MSS/TP53 did not overlap with the TCGA EBV subtype, as EBV^+^ tumors represented a small proportion of samples in the MSS/TP53^+^ subtype.

Possible reasons for the partial overlap of these two classifications include differences related to the patient population (Korea in ACRG vs. USA and Western Europe in TCGA), tumor sampling (predominantly intestinal diffuse type in ACRG), and technological platforms (six distinct molecular platforms in TCGA including exome sequencing, copy number analysis, methylation, miRNA and mRNA expression vs. reliance upon mRNA expression in ACRG). Despite these differences, these two classification schemes not only clarified and simplified the genomic and epigenomic heterogeneity of GC, but also revealed distinct salient genomic features among gastric cancer subtypes linked to clinical outcomes. These molecular classification systems of GC lay the groundwork for targeted therapies, patient stratification for clinical trials and treatment, and improved prognostication.

## 4. Molecular Profiling of Gastric Carcinoma: Therapeutic Targets and Predictive Biomarkers

Complementary to molecular classification of GC, analysis of molecular profiles of tumors collected from individual patients using a multi-platform approach has led to identification of targets for therapy as well as biomarkers that may be predictive of tumor response to treatment.

### 4.1. Therapeutic Targets

Molecular profiling of tumors including GC has been employed with the hope of identifying actionable and predictive biomarkers. In one study, 666 specimens of GC were analyzed by immunohistochemistry, in-situ hybridization, and genomic DNA sequencing [42]. Some of the analyzed molecules included ribonucleotide reductase regulatory subunit M1 (RRM1), *O*-6-methylguanine-DNA-methyltransferase (MGMT), phosphatase and tensin homolog deleted on chromosome ten (PTEN), topoisomerase (TOP), thymidine synthase (TS), and excision repair cross-complementing 1 (ERCC1). 

Negative expression of three non-NCCN (National Comprehensive Cancer Network) compendium actionable targets including RRM1 (62%), MGMT (45%), and PTEN (58%) was identified in more than 40% of the tumor specimens. These data suggest potential sensitivity to gemcitabine, temozolomide, or PI3K inhibitors, respectively. Negative RRM1 expression is associated with higher response rates to gemcitabine-based chemotherapy regimens [59,60,61]. Therefore, stratifying patients based on RRM1 expression may increase the likelihood of gemcitabine efficacy. 

In the HER2-positive cohort, co-expression of TOP2A occurred most frequently (93%), suggesting potential sensitivity to a combined anthracycline/trastuzumab approach to treatment. Furthermore, 50% of patients demonstrated possible benefit from a combination of trastuzumab with 5FU/capecitabine based on concurrent low TS, 53% with irinotecan (high TOPO1), 63% with cisplatin (low ERCC1) and 55% with gemcitabine (low RRM1). 

### 4.2. Predictive Biomarkers

The potential of these biochemical markers to predict treatment response of tumors to chemotherapy was examined. Molecular profiling of tumor specimens from 27 patients with gastroesophageal carcinoma was conducted by the Caris Molecular Intelligence^®^ service (Phoenix, AZ, USA). These included eleven GC, nine EGJC, and seven esophageal carcinoma (EC) [13]. The frequencies of actionable targets (Table 4) and mutations including *TP53* (33%), *APC* (7.4%), *SMAD4* (7.4%), and *PIK3CA* (7.4%), were consistent with those in a cohort of 666 specimens of GC [42]. 

In several cases, the PFS based on tumor molecular profile (MP) was compared to that on therapy prior to molecular profiling. A ratio of PFS-MP to PFS prior to MP greater than 1.3 is considered clinically significant. As shown in the three cases in Table 5, the ratio of PFS on MP-based treatment to PFS on treatment prior to molecular profiling exceeds 1.3, suggesting the potential value of MP in guiding selection of individualized therapy [13,62]. These results support further investigation using large sets of data from patients to correlate treatment response with tumor MP, and testing the hypothesis that tumor MP guides the selection of optimal therapeutic regimen for individualized treatment.

## 5. Conclusions and Perspectives

Systemic treatment plays important roles in the multi-disciplinary management of gastric carcinoma. Cytotoxic drugs, targeted agents, and immunotherapeutics have been shown to provide clinical benefit though to a limited extent. With the exception of trastuzumab for HER2-amplified and PD-L1-expressing GC, a clinical tool to predict the treatment response and outcomes of the currently used systemic therapy is lacking. Moreover, tumor heterogeneity and molecular evolution of tumor during treatment contribute to therapeutic resistance. Clinically tested and validated biomarkers for predicting tumor response to systemic treatment will be needed for patients to derive maximal benefit and avoid unnecessary toxic side effects.

Molecular classification and profiling of GC generate potential targets for therapy as well as prognostic and predictive biomarkers. The TCGA and ACRG data have not only revealed the molecular and etiologic differences across the various subtypes of GC, but also yielded many potentially targetable genomic changes. In addition, molecular profiling of GC by analysis of proteomics [63,64,65] and microRNA [66] as well as detection of circulating tumor DNA in plasma and exosomes of patients with GC [67] have been reported. These platforms may help identify therapeutic biomarkers and targets that are complementary to tumor molecular profiling by genomic and immunohistochemical analyses as described above. 

Development of therapeutic agents targeting some of those molecular alterations as defined to the subtyping and profiling of GC are undergoing pre-clinical and clinical investigation. Identification and validation of prognostic and predictive biomarkers by correlation of molecular profiles of tumors with clinical outcomes such as tumor response, progression-free survival, and overall survival are indicated. Future studies aiming to identify and validate predictive tumor biomarkers through molecular profiling in large data sets are indicated. Results of these studies are expected to facilitate selection of optimal chemotherapy regimen individualized for the patients, and the development of novel targeted therapies.

While the molecular data brings the possible hope of developing precision therapies, many challenges must be overcome to fully understand and realize their clinical impact. First, it is imperative to design and implement clinical trials that take into account the molecular heterogeneity across the various subtypes of GC and develop protocols specific for each of these entities. The Personalized Antibodies from Gastro-Esophageal Adenocarcinoma (PANGEA) “umbrella trial” is one such innovative trial in which patients are assigned to different treatment arms by matching the molecular characteristics of a single tumor type to a specific drug [68,69]. Considering that the tumor mutational profiles can evolve over time and in response to treatment, the adaptive design of this trial, which allows modifications of some aspects to be made while the trial is ongoing, would be very beneficial by matching the right drugs to the right patients in a time-sensitive fashion. 

Secondly, targeted therapy guided by molecular profiling will need to be tested in patient-derived tumor xenografts (PDX) and genetically-engineered mouse (GEM) models. The development and characterization of these realistic model systems represent the complex molecular heterogeneity of GC. They will be helpful for validating the genomic alterations in the molecular subtypes of GC and facilitating drug and biomarker development. Finally, development of novel therapies combining immunotherapeutics, cytotoxic chemotherapeutic agents, and molecularly-targeted therapeutics is expected to offer durable clinical benefits and maximize survival in patients with GC.

By integrating the various molecular and clinical data, we hope to develop strategies that will enable clinicians and scientists to better characterize and classify these tumors, develop targeted therapies, and identify prognostic and predictive biomarkers for achieving the goal of precision therapy in patients with this malignant disease.

## Figures and Tables

**Table 1:** Major phase III clinical trials of first-line cytotoxic agents in metastatic/advanced gastric carcinoma.

Treatment	Patients (*n*)	RR (%)	PFS (months)	OS (months)	Reference
CF vs. DCF	224 vs. 221	25 vs. 37	3.7 vs. 5.6 (* *p* < 0.001)	8.6 vs. 9.2 (* *p* < 0.02)	[22]
ECF vs. ECX vs. EOF vs. EOX	263 vs. 250 vs. 245 vs. 244	38 vs. 41 vs. 40 vs. 47	6.2 vs. 6.7 vs. 6.5 vs. 7.0 (NS)	9.9 vs. 9.9 vs. 9.3 vs. 11.2 (NS)	[23]
5-FU + LV + cisplatin vs. 5-FU + LV + oxaliplatin	112 vs. 106	25 vs. 34	3.9 vs. 5.8 (NS)	8.8 vs. 10.7 (NS)	[24]
Cisplatin + 5-FU vs. Cisplatin + S-1	508 vs. 521	31 vs. 29	5.6 vs. 5.3 (NS)	7.9 vs. 8.6 (NS)	[25]

CF, cisplatin/5-fluorouracil (5-FU); DCF, docetaxel/cisplatin/5-FU; ECF, epirubicin/cisplatin/5-FU; ECX, epirubicin/cisplatin/capecitabine; EOF, epirubicin/oxaliplatin/5-FU; EOX, epirubicin/oxaliplatin/ capecitabine; LV, leucovorin; NS, not statistically significant; OS, overall survival; * *p* < 0.05, statistically significant; PFS, progression-free survival; RR, response rate.

**Table 2:** TCGA molecular subtypes of gastric carcinoma and the associated targets and targeted agents.

Subtypes	Targets	Targeted Agents
EBV	PIK3CA	Idelalisib, Taselisib
	PD-L1/L2	Pembrolizumab, Nivolumab, Durvalumab, Avelumab, Atezolizumab
MSI	MLH1 silencing	Pembrolizumab, Nivolumab, Durvalumab, Avelumab, Atezolizumab
	PIK3CA	Idelalisib, Taselisib
	EGFR	Erlotinib, Gefitinib
	ERBB2	Trastuzumab
	ERBB3	Pertuzumab
	PD-L1	Pembrolizumab, Nivolumab, Durvalumab, Avelumab, Atezolizumab
CIN	EGFR	Erlotinib, Gefitinib
	VEGFA	Bevacizumab, Ramucirumab
	CCNE1, CCND1, CDK6	Palbociclib, Ribociclib, Abemaciclib
GS	RHOA	-
	CLDN18	-

CDK, cyclin-dependent kinase; CCND, cyclin D; CCNE, cyclin E; CIN, chromosomal instability; CLDN, claudin; EBV, Epstein–Barr virus; EGFR, epidermal growth factor receptor; GS, genomically stable; MLH1, MutL homolog 1; MSI, microsatellite instability; PD-L1/L2, programmed death ligand 1/ligand 2; PIK3CA, phosphatidylinositol-4,5-bisphosphate 3-kinase catalytic subunit alpha; VEGF, vascular endothelial growth factor

**Table 3:** Distribution of key genomic alterations across molecular subtypes of gastric carcinoma from TCGA and ACRG data.

Genetic Alteration	TCGA				ACRG			
	MSI	EBV	GS	CIN	MSI	MSS/EMT	MSS/TP53^+^	MSS/TP53^−^
*HER2* amp	0	12	3	22	0	0	3.0	17.4
*HER2* mut	11	4	3	3	16.3	2.8	0	4.7
*MET* amp	2	0	0	7	1.6	0	3.0	3.5
*PIK3CA* amp	3	8	2	7	0	0	0	1.1
*PIK3CA* mut	42	77	10	3	32.6	8.3	16.9	4.7
*KRAS* mut	23	4	9	5	23.3	0	8.5	3.5
*RHOA* mut	5	8	14	2	0	2.8	6.8	3.5
*CDH1* mut	8	0	34	3	7.0	2.8	1.7	3.5
*FGFR2* amp	0	0	7	7	0	4.9	3.0	1.2
*BRAF* mut	22	8	0	0	11.6	2.8	1.7	3.5
*ALK* mut	9	0	5	2	16.3	0	0	2.4
*ARID1A* mut	84	54	16	9	44.2	13.9	18.6	5.9
*TP53* mut	39	4	14	70	25.6	33.3	23.7	60
*PTEN* mut	25	15	2	1	14	5.6	3.4	3.5
*MTOR* mut	30	4	3	1	14	0	1.7	3.5
*APC* mut	36	0	3	12	16.3	2.8	15.3	8.2
*FBXW7* mut	34	0	5	1	16.3	2.8	1.7	2.4
*SMAD4* mut	8	12	9	7	4.7	2.8	8.5	2.4

MSI, microsatellite instability; EBV, Epstein–Barr virus; GS, genomically stable; CIN, chromosome instability; MSS, microsatellite stability; EMT, epithelial-mesenchymal transition; amp, amplification; mut, mutation; Numbers refer to % of samples with the genomic alteration.

**Table 4:** Frequency of actionable targets tested by immunohistochemistry along with the associated therapeutic agents.

Biomarker	Number of Specimens (%)	Beneficial Agents
TS (−)	19 (70.4)	Fluorouracil, Capecitabine
TOPO1 (+) *	16 (59.3)	Irinotecan, Topotecan
PTEN (−)	11 (40.7)	Trastuzumab, anti-EGFR
ERCC1 (−) *	11 (40.7)	Cisplatin, Carboplatin, Oxaliplatin
TOP2A (+) *	11 (40.7)	Doxorubicin, Epirubicin
RRM1 (−)	10 (37.0)	Gemcitabine
MGMT (−)	9 (33.3)	Temozolomide, Dacarbazine
TUBB3 (−)	8 (29.6)	Docetaxel, *nab*-paclitaxel, paclitaxel
cMET (+)	7 (25.9)	Anti-MET
TLE3 (+)	6 (22.2)	Docetaxel, Paclitaxel
SPARC Mono (+)	5 (18.5)	*nab*-Paclitaxel
SPARC Poly (+)	4 (14.8)	*nab*-Paclitaxel
HER2 (+) *	4 (14.8)	Trastuzumab, Lapatinib
PGP (−) *	4 (14.8)	Taxane

* Biomarker with associated agent on the National Comprehensive Cancer Network (NCCN) compendium. ERCC1, excision repair cross-complementation group 1; HER2, human epidermal growth factor receptor 2; cMET, hepatocyte growth factor receptor; MGMT, *O*-6-methylguanine-DNA methyltransferase; PGP, p-glycoprotein; PTEN, phosphatase and tensin homolog; RRM1, ribonucleotide reductase subunit M1; SPARC, secreted protein acidic and rich in cysteine; TLE3, transducin-like enhancer of split 3; TOPO1, topoisomerase 1; TOP2A, topoisomerase 2A; TS, thymidylate synthase; TUBB3, tubulin beta 3. The data are modified from [13].

**Table 5:** Progression-free survival on molecular profile-matched therapy vs. on prior therapy.

Biomarker	Method	Beneficial Agent	Treatment Prior to MP	MP-Based Treatment	PFS Ratio
HER2/Neu amplification	FISH, IHC	Trastuzumab	Docetaxel + Irinotecan PFS = 2.2 months	Trastuzumab + Docetaxel + Irinotecan PFS = 6.3 months	2.9
Topoisomerase 1 positive	IHC	Irinotecan, Topotecan	Epirubicin + Oxaliplatin + Capecitabine PFS = 2.3 months	Docetaxel + Irinotecan PFS = 4.5 months	2.0
SPARC Monoclonal positive	IHC	*nab*-Paclitaxel	Docetaxel + Irinotecan PFS = 1.9 months	Gemcitabine + *nab*-Paclitaxel PFS = 3.6 month	1.9

FISH, fluorescent in situ hybridization; HER2, human epidermal growth factor receptor 2; IHC, immunohistochemistry; MP, molecular profile; PFS, progression-free survival; PFS ratio, PFS on MP-matched therapy vs. PFS on prior therapy; SPARC, secreted protein acidic and rich in cysteine.
